# Multiple Intraosseous Calvarial Hemangiomas Mimicking Metastasis from Renal Cell Carcinoma

**DOI:** 10.1155/2008/176392

**Published:** 2007-11-12

**Authors:** Rohit Malde, Tim Moss, George Malcolm, Tim Whittlestone, Amit Bahl

**Affiliations:** ^1^Department of Clinical Oncology, Bristol Haematology and Oncology Centre, Bristol BS2 8ED, UK; ^2^Department of Neuropathology, Frenchay Hospital, Bristol BS16 1LE, UK; ^3^Department of Neurosurgey, Frenchay Hospital, Bristol BS16 1LE, UK; ^4^Department of Urology, Bristol Royal Infirmary, Bristol BS1 3NU, UK

## Abstract

Renal cell carcinomas are known to metastasise to the bones in the form of lytic lesions. However, not all osteolytic lesions in patients with renal cell carcinoma are metastatic in nature. The report describes the case of a 68-year old lady who was diagnosed with a renal cell carcinoma 3 and half years back and treated with radical nephrectomy along with excision of an inferior vena cava tumour thrombus. The tumour was completely excised and she remained disease free till date. Subsequently, multiple lytic lesions were detected incidentally on the cranial vault, which on biopsy demonstrated intraosseous hemangioma. Though it is well known that renal cell carcinomas can metastasise to the bones in the form lytic lesions, it is important for clinicians to remember a few other differentials, one of which would be an intraosseous hemangioma, which is a benign pathology. Many times patients would be treated as having metastatic disease merely on radiological findings. In this case report, there was a high index of radiological suspicion for metastases, however establishing diagnosis by biopsy prevented overtreatment in this instance.

## 1. INTRODUCTION

Renal cell carcinoma (RCC) accounts for approximately 3% of adult malignancies and 90–95% of neoplasms arising from the kidney [1]. Extension of the RCC into the renal vein and/or inferior vena cava (IVC) is present in about 4–15% of cases [2]. In patients with IVC involvement without metastases, an aggressive surgical approach in the form of radical nephrectomy with thrombectomy may be performed, as the 5-year survival of such patients can be as high as 68% [3, 4]. RCC can recur at any time after nephrectomy and usually metastasises via venous and lymphatic routes. The estimated risk of developing metastatic disease with T3 tumours is 33 to 43 percent at five years [5]. Bone is the second commonest site of metastasis occurring in 16–31% of patients, following lung metastasis, which is seen in 29–54% of patients following nephrectomy [5–7]. Bone metastasis is predominantly presented as lytic lesions in 71%, osteoblastic in 18%, and mixed variety in 11% of cases [8]. This case presents a lady who was detected to have multiple lytic lesions in the cranial vault, which was highly suspicious of metastatic disease on radiological investigations; however a biopsy revealed an intraosseous hemangioma.

## 2. CASE PRESENTATION

A 68-year-old lady presented to the accident and emergency with 5 episodes of sudden collapse/presyncopal episodes lasting for less than 30 seconds with complete and rapid recovery. These episodes were aggravated on standing or sitting and relieved on lying down. These episodes were not associated with any aura, seizures, palpitations, weakness, or incontinence. She also complained of gradual onset mild headache and feeling of dizziness over the past few days. This headache was dull aching and intermittent and relieved by analgesics. There was no history of any trauma, intoxication, or any febrile episode preceding current symptoms.

On examination, this lady was fully alert, conscious, and well oriented. All her vital parameters were normal. Her cardiovascular examination was normal with normal heart sounds and no murmurs were heard. Her blood pressure supine and upright were 138/77 and 128/83 mm Hg, respectively. A neurological evaluation revealed her Glasgow coma score of 15/15 with intact higher motor functions and no evidence of cranial nerve or sensorimotor deficit. Her remaining systemic examination was also essentially unremarkable.

On evaluation at the A & E, she was found to have blood glucose of 6.2 mmol/L. A full blood count, along with the electrolytes, and liver and kidney function tests were all within normal range apart from a low K^+^ = 2.8 mmol/L which was corrected. An initial electrocardiogram was also normal. She was later subjected to a 24-hour tape, which only revealed a few premature atrial complexes.

In her past medical history, she had a stable angina for the last 15 years and hypercholesterolemia for the last 7 years. She underwent cardiac catheterisation following an episode of angina 7 years ago and was detected to have normal coronary vessels. More interestingly, she had similar presyncopal episodes 2 years ago, when she was thoroughly evaluated in the form of a loop recorder which similarly had revealed frequent premature atrial complexes seen singly or as brief runs (3-4 beats). She had a history of hiatus hernia and diverticular disease diagnosed some 10 years back. Her regular medications included a diuretic, an antacid, and a beta-blocker antihypertensive.

From the oncology viewpoint, she was diagnosed to have renal cell carcinoma 3 and half years ago involving right kidney with extension into the renal vein and possibly into the IVC. She underwent right radical nephrectomy with IVC exploration and it was found that the tumour did not extend through the Gerota's fascia, however there was tumour extension through the renal veins, into the IVC extending up to the hepatic edge on palpation. The IVC was opened and the tumour thrombus excised in toto. She recovered well post-operatively and the histopathology showed a large tumour mass at the upper pole of the kidney measuring 9 × 5 × 4 cm distorting the renal capsule. The microscopy revealed a renal cell carcinoma of clear cell type (Fuhrman Grade 3) that did not penetrate the renal capsule; however there was invasion into the lumen of renal vein. She was thus pathologically staged as pT3b and kept under regular follow up. She had follow up CT scans of her chest, abdomen, and pelvis at 6 monthly intervals, which did not show any evidence of recurrence locally or in the form of distant metastasis.

**Figure 1:**
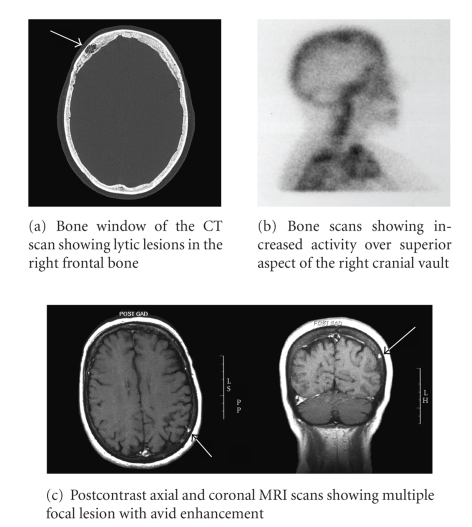
Radiology features of the lytic lesions.

**Figure 2:**
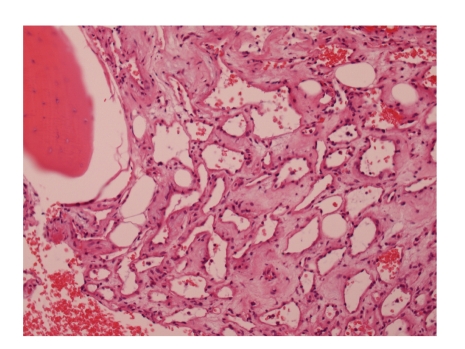
Histology demonstrating multiple tiny fragments of trabecular bone and marrow tissue being replaced by a mixture of fat, loose fibrous connective tissue, and thin walled multiple vascular channels which are suggestive of a benign intraosseous hemangioma.

She was admitted in the hospital and kept under observation, where she complained of another two presyncopal episodes for which no cause could be identified. In view of her past oncology history and current symptoms, a CT scan of the brain was performed which incidentally showed two lytic lesions in the right frontal bone, the largest measuring 3 cm in size. These were suspicious for metastasis (Figure 1(a)). There was, however, no intracranial abnormality. A myeloma screen in the form of protein electrophoresis, urinary Bence Jones protein was all negative. Though, the patient did not have any further presyncopal episodes, her scenario was discussed in the urology multidisciplinary team (MDT) meeting, where it was decided to evaluate these lesions further by requesting a magnetic resonance imaging (MRI) of her whole brain and a bone scan on an out patient basis. The bone scan revealed mildly increased isotope activity over the superior aspect of the right cranial vault probably consistent with an isolated metastasis. There was no pathological uptake elsewhere in the body (Figure 1(b)). Subsequently, an MRI scan of the whole brain was performed which demonstrated not only the two lesions seen in the right frontal bone, but in addition detected two further tiny lesions in the left posterior parietal bone. The epicentres of all these lesions were in the dipole of the cranial vault and following contrast enhancement these lesions showed an avid enhancement (Figure 1(c)). All the radiological findings were consistent with cranial vault metastasis. Her scenario was again discussed in the MDT, where the findings, though unusual, were more or less agreed to be due to metastasis. There were no imaging studies done of her cranial vault or brain at or before her oncological diagnosis. In view of her symptoms being not related to her vault lesions, an option of biopsying the lesion from the cranial vault was given to the patient, which she agreed to undergo. This lady thus underwent excision of right frontal skull lesion under stealth guidance followed by acrylic cranioplasty. The histopathology sections showed multiple tiny fragments of trabecular bone and marrow tissue that was replaced by a mixture of fat, loose fibrous connective tissue, and thin walled multiple vascular channels, mostly of small calibre. This was consistent with the appearance of a benign intraosseous hemangioma (Figure 2).

## 3. DISCUSSION

The unexpected finding of one or more radiolucencies in the skull is a finding that occurs not infrequently. Deciding the optimum management pathway for these is a dilemma. This case report describes the approach taken by our clinical team. Roughly, for every 100 patients undergoing some roentgenography of the skull at a tertiary hospital, about 40 would show calvarial translucencies. The majority of these would be clinically innocuous and ignored, however 1/6th of these (7% of all) would show lytic lesions of some concern (9). The incidence of hemangiomas occurring in such a fashion is similar to that of bone metastasis, occurring as 3% each of all such incidental findings. The presence of multiple lesions as commonly seen in both hemangiomas and metastasis makes the process of narrowing the differentials extremely difficult. Moreover, in accordance with the *law of parsimony*, in patients with multiple lesions, a single etiological factor is responsible for all the lesions in most cases.

Osseous hemangiomas are benign osseous malformed vascular lesions accounting for less than 1% of all primary bone neoplasms. They occur most frequently in the vertebral column (30–50%) and skull (20%), whereas involvement of other sites like the long bones, short tubular bones, and ribs is quite uncommon (10). They are two to three times more common in females than males. There are four histologic variants of hemangiomas, classified accordingly to the predominant type of vascular channel: cavernous, capillary, arteriovenous, and venous. These types can coexist. The cavernous hemangiomas most frequently occur in the skull whereas capillary hemangiomas predominate in the vertebral column. Hemangiomas are slow growing and malignant degeneration is virtually unknown.

Calvarial hemangiomas arise predominantly from the diplopic space and are usually located in the frontal and parietal regions. They are usually round, lytic lesions that may demonstrate a characteristic sunburst, radiating spoke wheel or web-like pattern of trabecular thickening (11). However, most often the findings are nonspecific and unhelpful in detecting these lesions. Despite the advances in diagnostic imaging, the angiomatous nature of the lesions can only be confirmed with histological diagnosis as the differentials in general may include Pagets disease, osteoma, bone metastasis, osseous lymphoma, multiple myeloma, aneurysmal bone cyst, primary osteosarcoma, and rarely, even intraosseous meningiomas.

Our patient who had initially presented with presyncopal attacks was thoroughly evaluated, but unfortunately a specific cause for her symptom could not be identified. A literature review of nearly 521 patients in a retrospective study showed the causes of syncope in patients without any structural heart disease to be autonomic nervous system (ANS) dysfunction (58.6%), syncope of cardiogenic origin (2.5%), or nonneurogenic hypotension (1.3%) and loss of consciousness of nonsyncopal origin (8.4%). The cause remains unknown in nearly 29.2% of the cases (12).

## 4. CONCLUSION

It is well known that bones are one of the common sites of metastasis from a renal cell carcinoma. However, not all osteolytic lesions of the bone are metastatic in nature. The clinician should consider other differentials like calvarial hemangiomas, which may mimic the neuroimaging finding of more ominous lesions. A histopathological verification of the diagnosis may help avoid unnecessary anticancer treatment.
